# Spatiotemporal Analysis of Electronic Cigarette Perception on Twitter/X Using Natural Language Processing

**DOI:** 10.21203/rs.3.rs-8981892/v1

**Published:** 2026-03-17

**Authors:** Zidian Xie, Jiamu Tang, Dongmei Li

**Affiliations:** University of Rochester Medical Center; University of Rochester; University of Rochester Medical Center

**Keywords:** E-cigarettes, Social media, Twitter/X, Deep learning model, Sentiment, Topic

## Abstract

**Background:**

Electronic cigarettes (e-cigarettes) have become popular in recent years, particularly among the youth and young adults. This study aims to examine the spatiotemporal patterns of online perception of e-cigarettes on Twitter/X.

**Methods:**

Through the Twitter API (Application Programming Interface), over 3 million e-cigarette-related tweets were collected from March 11, 2021, to March 14, 2023, using related keywords, such as “e-cigarette” and “vaping”. After data cleaning (such as removing duplicates and retweets) and filtering, 2,140,439 non-commercial tweets were identified. Two human coders independently hand-coded 300 randomly selected tweets regarding relevance (yes or no), sentiment (positive, negative, or neutral), and whether the Twitter user is a likely e-cigarette user (yes or no). An additional 2,000 randomly selected tweets were single-coded. The labeled 2,300 tweets were used to fine-tune a pre-trained RoBERTa (Robustly Optimized BERT) model, which achieved good performance (F1 scores > 0.7). The Latent Dirichlet Allocation (LDA) method was used to identify the major topics in tweets with either positive or negative sentiment.

**Results:**

We observed a noticeable increase in the number of e-cigarette-related tweets, especially in the UK and Australia, during the study period. Nearly half of the tweets (49.7%, 1,063,317/2,140,439) were neutral. The proportion of tweets with a positive sentiment toward e-cigarettes was higher than that with a negative sentiment, at 27.0% vs. 23.3%. Except for Australia, in the US and UK, especially Canada, there were more positive tweets than negative ones. There was a rising trend in the proportion of tweets with a negative sentiment in the UK and Australia. Additionally, e-cigarette Twitter users were more likely to hold a positive sentiment toward e-cigarettes than non-users, 41.19% vs. 9.74%. Positive topics framed vaping as a desirable, emotionally driven alternative that supports smoking cessation, whereas negative topics emphasized health risks, youth harm, environmental concerns, and calls to quit despite perceived reduced harm.

**Conclusions:**

Online perceptions of e-cigarettes on Twitter varied over time and across different countries. E-cigarette users and non-users held different sentiments toward e-cigarettes. Findings from this study provide timely monitoring in online perception of e-cigarettes on social media, offering valuable guidance for future tobacco regulations.

## INTRODUCTION

Electronic cigarettes (e-cigarettes) are the most commonly used tobacco products in US middle and high school students, according to the 2024 National Youth Tobacco Survey [1]. The prevalence of current e-cigarette use (vaping) is 7.8% among high school students and 3.5% among middle school students in 2024 [2]. Although the long-term health effects of vaping are largely unknown, experimental and clinical studies have reported the acute health effects of vaping, including toxicity in the respiratory and cardiovascular systems [3]. Epidemiology studies associated vaping with respiratory disorders, such as wheezing, asthma, and chronic obstructive pulmonary disease, and mental health issues [4–10].

Regulatory policies for e-cigarettes vary across countries. In 2009, the U.S. began regulating e-cigarettes as tobacco products, and in 2016, the US Food and Drug Administration (FDA) expanded its authority to regulate e-cigarettes as tobacco products [11]. In 2020, to prevent youth access to flavored e-cigarettes, the US FDA implemented a flavor enforcement policy to restrict the sale of all unauthorized flavored cartridge-based e-cigarettes other than tobacco and menthol flavors [12]. The e-cigarette products in Canada have a nicotine concentration cap of 20 mg/ml, with federal laws restricting their advertising, mandating plain packaging, and prohibiting their sales to minors [13]. In the UK, e-cigarettes are regulated as a less harmful smoking cessation tool with restrictions on nicotine strength (20mg/ml), tank capacity (2ml), and advertisement [14]. Australia has the world’s strictest e-cigarette policies, with e-cigarettes classified as prescription-only medicines in 2021 and announced plans in 2023 to ban disposable vapes and restrict flavors to curb youth uptake of e-cigarettes [15]. Regulatory policies on e-cigarettes can have a significant impact on the perception and prevalence of e-cigarette use. For example, the UK government’ 2024 vaping policy announcement exacerbated negative harm perceptions of e-cigarettes [16]. Furthermore, cross-national differences in e-cigarette regulatory policies may shape public perceptions and influence the prevalence and patterns of e-cigarette use.

With the continuing battle between tobacco regulatory policies and aggressive industry marketing, monitoring public perceptions of e-cigarettes is critical, as these perceptions may directly influence the prevalence of e-cigarette use. Twitter (now X) data have been extensively used to examine online perceptions and discussions of e-cigarettes, as well as how regulatory policies influence these perceptions and discussions [17–21]. Previous studies showed that Twitter users’ sentiments toward e-cigarettes became significantly more negative following announcements by New York State and the US FDA regarding bans on the sale of flavored e-cigarettes, excluding menthol and tobacco flavors [18, 19]. Subsequent work further evaluated the impact of New York State’s comprehensive flavor ban—which prohibits the sale of all flavored vapor products except tobacco and other authorized flavors—and found increased prevalence of discussions related to youth, nicotine products, and quitting behaviors using Twitter data from February to November 2020 [21]. Furthermore, one study reported that discussions surrounding vaping cessation became more prominent following the FDA’s announcement and implementation of the flavor enforcement policy [17]. Beyond the U.S. context, one prior study leveraged Twitter data to examine public perceptions of e-cigarette prescription policies in Australia and the UK, where e-cigarettes are regulated as medical products [20]. This study found that UK Twitter users expressed more positive attitudes toward the prescription policy than Australian users. Specifically, tweets with positive sentiment in the UK more frequently framed e-cigarettes as a smoking cessation aid, whereas positive tweets in Australia focused on health effects. In contrast, negative sentiments emphasized economic consequences in the UK and concerns about hindering smoking cessation in Australia [20].

Given the rapid evolution of e-cigarette marketing and regulatory policies across countries, it is critical to continuously update and monitor online perceptions and discussions of e-cigarettes on social media platforms such as Twitter/X across both temporal and geographic dimensions. Using Twitter/X data collected from March 2021 to March 2023, this study examined online perceptions and discussions of e-cigarettes in the US, Canada, the UK, and Australia using deep learning–based natural language processing approaches. By integrating temporal trends, cross-country comparisons, and user-level e-cigarette use status, this study provides timely evidence to inform the development and evaluation of e-cigarette regulatory policies in the US and other countries.

## METHODS

### Data collection

Through Twitter/X streaming API (Application Programming Interface), e-cigarette-related English tweets were collected between May 3, 2021, and March 14, 2023, using keywords directly related to electronic cigarettes, such as “e-cig” and “vape”[22, 23]. In total, 6,940,065 tweets related to e-cigarettes were collected.

### Data pre-processing

Duplicate tweets and retweets were removed, resulting in 3,045,114 unique tweets. To identify organic discussions, we removed tweets with commercial content (e.g., e-cigarette advertisements from vape shops and small businesses) using keyword filtering within usernames and tweet content (e.g., “deal,” “sale,” “promo”) [18, 21, 24, 25]. This process resulted in 2,575,607 non-commercial tweets. In addition, we performed geoprocessing to identify tweets from four countries: the United States, the United Kingdom, Australia, and Canada.

### Human-guided deep learning language models

In this study, considering some tweets might not be directly related to e-cigarettes even though they contain e-cigarette-related hashtags, first, we want to determine if the tweet is related to e-cigarettes (Relevancy). Next, we want to determine the sentiment of each tweet toward e-cigarettes/vaping, including positive, negative, or neutral sentiment. In addition, we want to check if the tweet was from a potential e-cigarette user (vapers) or a non-e-cigarette user (non-vapers). To do this, we manually labeled a random sample of 2,601 tweets to create a reliable dataset for classifier training. Firstly, two human coders independently labelled 300 tweets (randomly selected from the dataset). However, the Cohen’s kappa values were below the threshold of 0.70, indicating relatively low inter-rater agreement. Then, after resolving the discrepancies among the first 300 tweets through the discussion in the group of three members, a second set of 300 tweets was randomly selected and hand-coded independently by the same two coders. For the second round, the kappa value for relevancy reached 0.80, the kappa value for the sentiment achieved 0.72, and the kappa value for the user type (vapers or non-vapers) reached 0.74, indicating substantial inter-rater agreement. Then, two trained human coders single-coded an additional 2001 tweets. In total, 2,601 tweets were manually coded.

For classification, in this study we will apply RoBERTa (Robustly Optimized BERT Pretraining Approach), transformed-based models [26]. RoBERTa’s fine-tuning capabilities allow for accurate classification across nuanced categories, making it suitable for sentiment analysis and context-based filtering in social media datasets. We split our labelled dataset (2,601 labelled tweets) into 80% for training and 20% for testing to ensure robust model performance and reliable evaluation. Using RoBERTa’s pre-trained language model, we fine-tuned each classifier for our specific tasks to leverage its advanced language representation on Twitter data. To address imbalances in the dataset, we applied sampling techniques, including oversampling and undersampling, particularly when certain categories (e.g., positive vs. neutral sentiment) were underrepresented. Regarding e-cigarette relevance, the model’s F1 score reached 0.87. For sentiment towards e-cigarettes, the model achieved an F1 score of 0.82. The model’s F1 score for identifying vapers was 0.81. Therefore, the RoBERTa models demonstrated good model performance. These models were used to label all non-commercial tweets. Finally, only 2,140,439 tweets were identified as relevant to e-cigarettes.

Given the complexity of determining user identity from an individual tweet, a two-step classification process was employed to enhance accuracy and robustness. First, a RoBERTa-based user type classification model was applied at the tweet level to determine whether a given tweet was posted by an e-cigarette user (vaper). Second, user IDs were aggregated, allowing multiple tweets from the same user to be analyzed collectively. This aggregation ensured a more reliable classification of individual users as vapers or non-vapers, rather than making inferences based on isolated tweets.

### Topic modeling

To uncover prominent themes within public discourse on e-cigarettes, we conducted topic modeling using Latent Dirichlet Allocation (LDA), a generative probabilistic model well-suited for large text datasets [27]. LDA identifies latent topics within a corpus by clustering terms based on co-occurrence patterns, making it highly effective for grouping related themes within tweets that express complex sentiments. Its flexibility and effectiveness in distinguishing nuanced topics across different text samples made it an ideal choice for exploring prevalent themes in our dataset. To ensure consistent and meaningful topic extraction, we standardized the dataset through several preprocessing steps. We converted all text to lowercase, lemmatized terms, and removed stop words, such as personal pronouns and prepositions, using spaCy and the Natural Language Toolkit (NLTK). This preprocessing was crucial for minimizing noise and maximizing coherence within each topic. Additionally, we identified frequent bigrams (e.g., “quit vaping”) and trigrams (e.g., “vaping health risks”) using the Gensim package, treating these common phrases as single terms within the LDA model. This setup enhanced the model’s ability to detect clear, contextually relevant topics. To determine the optimal number of topics, we calculated coherence scores for different numbers of topics and selected the one with the highest score. The coherence score quantitatively evaluates the interpretability of topics, ensuring that each topic represents a distinct theme within the data. To understand potential underlying reasons, we performed LDA topic modeling on e-cigarette-related tweets with either positive or negative sentiment.

## RESULTS

### Identification of e-cigarette-related tweets

**Figure 1:**
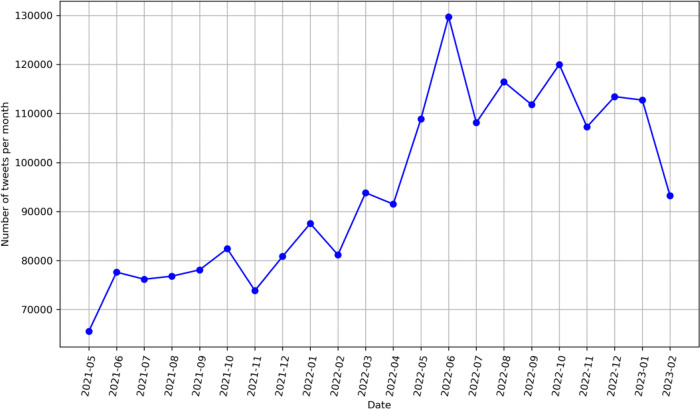
Temporal trend in the prevalence of e-cigarette-related English tweets

From May 3, 2021, to March 14, 2023, we have identified a total of 6,940,065 English tweets using keywords related to e-cigarettes. Among them, there were 2,140,439 unique non-commercial tweets related to e-cigarettes. As shown in Figure 1, the number of tweets per month exhibited a steady increase over time in our studying period with a notable peak in June 2022 and a slight decrease in February 2023. Based on the geolocation information shared on the tweets, we have identified 430,929 tweets from the US, 90,811tweets from the UK, 43,453 tweets from Australia, and 39,552 from Canada. The rest of tweets were either from other countries or no valid geolocation information. Similarly, the mentions of e-cigarettes on Twitter showed an overall increasing trend in the US, UK, and Australia except in Canada (Supplemental Figure 1).

### Online sentiments toward e-cigarettes on Twitter/X

Sentiment analysis was conducted to classify tweets into positive, negative, or neutral sentiment towards e-cigarettes. Among the 2,140,439 tweets related to e-cigarettes, 27.00% (578,016 tweets) expressed a positive sentiment towards e-cigarettes, 49.68% (1,063,317 tweets) were categorized as neutral, and 23.32% (499,106 tweets) expressed a negative sentiment (Table 1). While the proportion of tweets with the positive and negative sentiment towards e-cigarettes were similar in the US and UK, the proportion of tweets with a positive sentiment was higher than those with a negative sentiment in Canada, 29.09% vs. 23.94% (P-value < 0.01). In contrast, the proportion of tweets with a negative sentiment was higher than those with a positive sentiment in Australia, 30.39% vs. 23.76% (P-value < 0.01).

**Figure 2:**
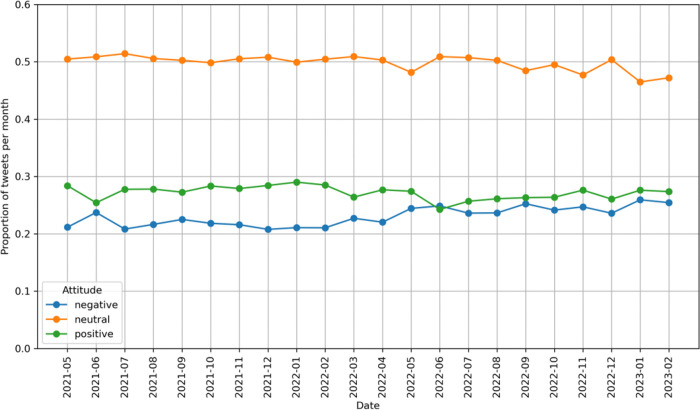
Relative prevalence of tweets with different sentiments toward e-cigarettes over time.

As shown in Figure 2, the relative prevalence of tweets with different sentiments remains relatively constant during the studying period, except the proportion of tweets with the positive sentiment showing a decreasing trend while an increasing trend for negative tweets in June 2022. In addition, the difference in the prevalence of tweets between the positive and negative sentiment became smaller since June 2022. We observed similar trends in the US and Canada (Supplemental Figure 2). However, in the UK, while there were more tweets with a positive sentiment than those with a negative sentiment before June 2022, the proportion of tweets with a positive sentiment became less than those with a negative sentiment after June 2022. In Australia, the number of tweets with either a positive or negative sentiment were similar before June 2022. However, there were more tweets with a negative sentiment than those with a positive sentiment after June 2022.

### Online perception of e-cigarettes between vapers and non-vapers

**Figure 3:**
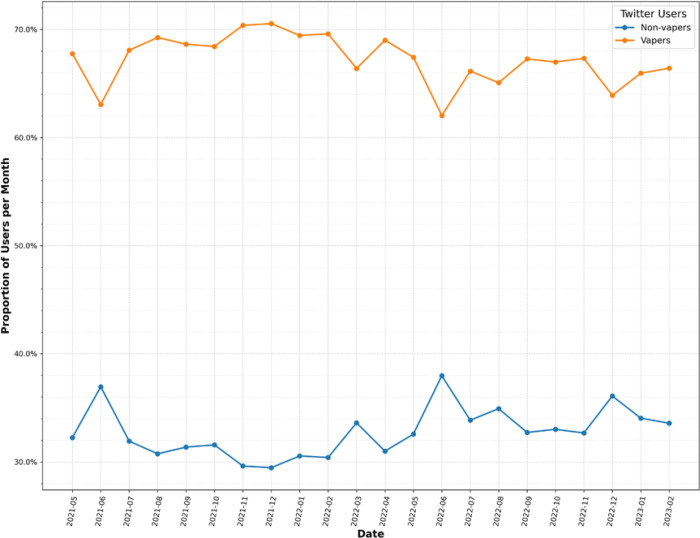
Relative prevalence of vapers and non-vapers among Twitter users posting e-cigarette-related tweets.

Among e-cigarette-related tweets, 603,600 unique Twitter users (57.28%) were identified as potential e-cigarette users (vapers), and 450,255 users (42.72%) were categorized as non-e-cigarette users (non-vapers). As shown in Figure 3, among Twitter users posting e-cigarette-related tweets, the relative prevalence of vapers was dominant over time, ranging from 60% to 70%. Furthermore, the prevalence of vapers showed a slight decrease over time, which reached the lowest level in June 2022. While the relative prevalence of vapers remains constant in the US and Canada, the relative abundance of vapers showed a clear decreasing trend over time in the UK and Australia (Supplemental Figure 3).

By examining the online perception of e-cigarettes on Twitter/X, vapers were more likely to exhibit a positive sentiment than non-vapers, 41.19% vs. 9.74% (Table 1). In contrast, they were less likely to hold a negative sentiment than non-vapers, 11.85% vs. 37.28%. By comparison, the proportion of tweets with a positive sentiment was the highest in Canada (29.09%), followed by the UK (27.93%), US (26.20%), and Australia (23.75%). In addition, Twitter users from Australia were more likely to hold a negative sentiment towards e-cigarettes (30.39%), followed by the UK (27.29%), US (25.46%), and Canada (23.84%). We observed similar patterns for vapers and non-vapers between these countries (Table 1).

### Online discussion about e-cigarettes on Twitter/X

To further understand the potential underlying reasons for different sentiments toward e-cigarettes on Twitter/X, main topics were identified from tweets with an either positive or negative sentiment (Supplemental Table 1). As shown in Table 2, four main topics have been identified from e-cigarette-related tweets with a positive sentiment. Two most popular topics included topic 3 “The desire for vaping” (32.77%) and topic 4 “Emotional triggers and routine user of e-cigarettes” (32.93%). Among tweets with a positive sentiment, 22.79% of them held a notion that vaping can help with smoking cessation, and 11.50% perceived vaping as a better choice than smoking. Compared to other three countries, positive tweets from the US were less likely to perceive e-cigarettes as a better choice than smoking or helping with smoking cessation (Table 2). Among nine main topics identified from tweets with a negative sentiment, topic 7 “Urge to quit vaping due to health risks and addiction” was the most popular one (22.18%), followed by topic 4 “Irresponsible vaping behaviors in public and youth settings” (18.08%) and topic 3 “Bad experiences with vaping” (16.24%). Some tweets (8.14%) acknowledged that e-cigarettes are not risk-free. Negative tweets from Australia were more likely to perceive vaping being bad for youth (15.81% vs. 8.06%) and e-cigarettes not being risk-free (19.98% vs. 8.14%), but less likely to concern its environmental impact (6.20% vs. 10.27%) than the overall.

By examining temporal trends in the proportion of topics with either a positive or negative sentiment, among tweets with a positive sentiment, topic 2 showed a peak while topic 4 showed a decrease in June 2022 (Supplemental Figure 4). At the same time, we observed that topic 6 showed an obvious peak and topic 4 showed a decrease in June 2022 among tweets with a negative sentiment (Supplemental Figure 5).

## DISCUSSION

In this study, by examining e-cigarette-related posts on Twitter/X, we provided a comprehensive understanding of online perception and discussions of e-cigarettes temporally and geographically. While there was an overall increase in the mentions of e-cigarettes on Twitter/X during the study period, there was an obvious peak in June 2022, especially in the US, which corresponds with the announcement of JUUL ban in the US. Although the proportion of tweets with a positive sentiment was slightly higher than those with a negative sentiment overall, the proportion of tweets with a positive sentiment showed a gradual decreasing trend, especially in the UK and Australia. Further analysis of e-cigarette-related tweets with either positive or negative sentiments, we have identified several main topics, which provide important insights about potential reasons underlying different sentiments.

We observed a gradual upward trend in discussing e-cigarettes on Twitter/X during our study period with a significant spike occurred in June 2022, especially in the US, which coincides with major JUUL-related policy decisions in the United States. On June 23, 2022, the US FDA issued marketing denial orders (MDOs) to all JUUL products in the US, which prohibits the company selling and distributing these products in the US market [28]. While the JUUL products were dominant in the US e-cigarette market in 2018 and 2019 [29], these MDOs resulted into active online discussion around the policy change as well as the e-cigarettes, as evidenced by the peak in related posts on Twitter/X. These findings indicate that discussions on e-cigarettes can be event-driven, with major regulatory actions and policy debates serving as catalysts for increased public discourse.

In this study, nearly half of tweets did not show clear sentiment (labelled as neutral) towards e-cigarettes, they consist of informational-based content, such as policy updates, scientific studies, and product-related announcements, without strong personal opinions. The overall proportion of e-cigarette-related tweets with a positive sentiment was slightly higher than those with a negative sentiment towards e-cigarettes, 27.00% vs. 23.32%. By comparison, tweets from Canada were more likely to show a positive sentiment towards e-cigarettes than those from Australia, 29.09% vs. 23.76%. While the underlying reasons remain elusive, different regulatory policies and cultures in different countries might partly account for such differences [30]. Compared to Canada, Australia has a much more restrictive regulation on e-cigarettes, such as classifying e-cigarettes as prescription-only therapeutic products, which might lead to reduced acceptance and less favorable attitudes toward e-cigarettes. In contrast, the number of tweets with either a positive or negative sentiment was similar in either the US or the UK. This result suggests that the discourse of e-cigarette perception in the US and UK is relatively balanced, with strong advocacy for vaping as a harm reduction tool coexisting with concerns over youth vaping, addiction, and health concerns. The sentiment of tweets varied significantly across countries, potentially influenced by local regulatory policies, public health narratives, and societal perceptions of vaping. Furthermore, as expected, we showed that vapers were more likely to show a positive sentiment towards e-cigarettes than non-vapers on Twitter, which is similar across four countries. This divergence highlights that while vapers tend to frame e-cigarettes in a more favorable light, non-vapers are more likely to view vaping through a public health risk lens, particularly in countries with stringent policies. The polarization of opinions between vapers and non-vapers underscores the complexity of the e-cigarette debate, with strong advocacy on both sides of the discourse.

Our temporal trend showed that while the proportion of tweets with different sentiments remain relatively consistent during our studying period, the difference in relative abundance between tweets with a positive and negative sentiment became smaller since June 2022. Our results further showed an increase in tweets posted by non-vapers in June 2022, suggesting that the JUUL announcement stimulated broader public discussion, particularly among non-vapers, regarding the health effects of e-cigarettes. Notably, in the UK and Australia, the proportion of tweets with positive sentiment decreased, while that with negative sentiment increased, starting from June 2022, which might be partially driven by increasing concerns over e-cigarettes’ health effects and environmental impact following the announcement of DMOs for JUUL products in the US. Our study showed that tobacco regulatory policies (such as JUUL DMOs in the US) in one country can significantly affect public perception of e-cigarettes in other countries, possibly because Twitter users in those countries worry about the implementation of such policies in their own countries. This suggests that tobacco regulatory decisions in one country can influence global public discourse, leading to a significant shift in public perception of e-cigarettes. Overall, the dynamic nature of vaping discourse underscores the ongoing public debate surrounding its benefits, risks, and societal implications.

Among tweets with a positive sentiment toward e-cigarettes, besides most tweets showed a desire of vaping, nearly a quarter of tweets considered vaping as a smoking cessation strategy, which is more pronounced in the UK (34.19%). In the UK, e-cigarettes are regarded as an effective smoking cessation method and the medical products [31–33]. About one tenth of tweets considered vaping as a safer alternative to smoking, which was much higher in Australia (23.54%). Therefore, our results suggest that e-cigarettes are more likely to be considered as a safer alternative and effective smoking cessation method in the UK and Australia than the US and Canada, which is consistent with previous findings [34, 35]. Temporal trend showed that there was a peak in topic 2 “Vaping is a better choice than smoking” while there was a decrease in topic 4 “Emotional triggers and routine use of vaping” in June 2022, especially in the US, possibly driven by the announcement of JUUL MDOs in the US. The JUUL ban triggered surges in online discourse about e-cigarettes, with one side of the voice against the ban by emphasizing the benefits of vaping and the desire for e-cigarettes.

Common discussions for the negative sentiment towards e-cigarettes include health concerns of vaping, complaining about vaping in public settings, especially around schools. The topic 4, “Irresponsible Vaping Behaviors in Public and Youth Settings,” was relatively low in Australia, which might be due to Australia’s much stricter policy on school vaping [36]. At the same time, we observed that this topic was significantly lower in June 2022, which might be a consequence of the JUUL ban, leading to less public concern about vaping. Recognizing that e-cigarettes are not risk-free, topic 6 experienced significant spikes with the announcement of the FDA’s JUUL ban in June 2022. In addition, the prevalence of this topic was higher in Australia than in other countries, suggesting that e-cigarettes are more likely to be considered not risk-free in Australia. A region-specific discussion emerged around “Environmental Concerns Over Disposable Vapes”, particularly in the UK. Parliamentary debates in 2022 on banning single-use disposable e-cigarettes fueled public discourse about their ecological impact. While this topic was prominent in the UK, similar discussions were less prevalent in other countries.

This study has several limitations. First, the demographics of Twitter users differ slightly from those of the public, so our results cannot represent the general population. Second, although our deep learning language models achieved good performance, they are not 100% accurate, which could introduce biases. Third, given the lack of demographic data on Twitter users, we cannot determine the perception of e-cigarettes across different demographic groups, especially among youth, which should be further investigated in future studies. Fourth, there may be tweets from social bots, which could introduce noise into our results. Lastly, amid the ongoing battle between the e-cigarette industry and regulatory policy, public perception of e-cigarettes is evolving, requiring ongoing monitoring and timely updates.

## CONCLUSIONS

By analyzing millions of e-cigarette-related tweets, this study provided temporal trends and geographic comparisons on online perception of e-cigarettes. Public perception of e-cigarettes is evolving, especially with the development of tobacco regulatory policies. While perceptions of e-cigarettes were similar across countries, some differences persisted, which might be driven by differences in tobacco regulatory policies and societal perceptions. In addition, regulatory policies in one country can significantly affect public perception of e-cigarettes in other countries. More importantly, what we learned from one country about the impact of certain regulatory policies can provide valuable insights into the potential impact of similar policies in other countries. Together, while there are different voices about e-cigarettes, it is critical to effectively communicate with the public about the potential health effects of e-cigarettes to further protect public health, especially among the youth.

## Supplementary Material

Supplementary Files

This is a list of supplementary files associated with this preprint. Click to download.

• SupplementalTable1.docx

## Figures and Tables

**Table 1.** Online perception of e-cigarettes between different user groups.

	Overall			Vapers			Non-vapers		
	Pos	Neg	Neural	Pos	Neg	Neural	Pos	Neg	Neural
**All (vapers: 603,600; non-vapers: 450,255)**	27.00%	23.32%	49.68%	41.19%	11.85%	46.96%	9.74%	37.28%	52.98%
**US (vapers: 125,285; non-vapers: 93,341)**	26.20%	25.46%	48.34%	40.7%	12.5%	46.9%	10.2%	39.9%	50.0%
**UK (vapers: 23,785; non-vapers: 21,121)**	27.93%	27.29%	44.78%	46.5%	12.2%	41.3%	11.0%	41.0%	48.0%
**Canada (vapers: 10,856; non-vapers: 8,384)**	29.09%	23.84%	47.07%	45.6%	10.5%	43.9%	11.4%	38.2%	50.4%
**Australia (vapers: 9,187; non-vapers: 8,667)**	23.76%	30.39%	45.85%	43.6%	12.4%	44.0%	10.6%	42.3%	47.1%

Note: Pos, positive; Neg, negative.

**Table 2.** Main topics identified in e-cigarette-related tweets with a positive or negative sentiment.

Sentiment	Topic	All	US	UK	Canada	Australia
Positive	Topic 1: Vaping helps with smoking cessation	131,754 (22.79%)	26,331 (26.58%)	8,023 (34.19%)	3,110 (29.48%)	2,591 (27.46%)
	Topic 2: Vaping is a better choice than smoking	66,479 (11.50%)	13,670 (13.80%)	3,986 (16.99%)	1,777 (16.84%)	2,221 (23.54%)
	Topic 3: The desire for vaping	189,439 (32.77%)	33,835 (34.16%)	5,780 (24.63%)	3,002 (28.45%)	2,454 (26.01%)
	Topic 4: Emotional Triggers and Routine Use of e-cigarettes	190,339 (32.93%)	25,208 (25.45%)	5,675 (24.19%)	2,661 (25.22%)	2,169 (22.99%)
	Total	578,016 (100%)	99,044 (100%)	23,464 (100%)	10,550 (100%)	9,435 (100%)
Negative	Topic 1: Complaints about tobacco industry	14,482 (2.90%)	2,309 (2.40%)	372 (1.62%)	173 (2.02%)	207 (1.68%)
	Topic 2: Health risks of vaping	22,288 (4.47%)	4,693 (4.88%)	1,032 (4.51%)	467 (5.46%)	721 (5.86%)
	Topic 3: Bad experiences with vaping	81,075 (16.24%)	16,310 (16.95%)	3,915 (17.10%)	1,388 (16.24%)	1,752 (14.24%)
	Topic 4: Irresponsible Vaping Behaviors in Public and Youth Settings	90,251 (18.08%)	16,082 (16.71%)	3,229 (14.10%)	1,284 (15.02%)	1,385 (11.26%)
	Topic 5: Vaping is bad for youth	40,223 (8.06%)	9,027 (9.38%)	2,399 (10.48%)	864 (10.11%)	1,945 (15.81%)
	Topic 6: Vaping is safer than smoking but not risk-free	40,627 (8.14%)	10,371 (10.78%)	2,622 (11.45%)	1,071 (12.53%)	2,458 (19.98%)
	Topic 7: Urge to quit vaping due to health risks and addiction	110,683 (22.18%)	18,410 (19.13%)	4,766 (20.82%)	1,563 (18.28%)	1,928 (15.67%)
	Topic 8: Calls for quitting vaping due to health and social perceptions	48,212 (9.66%)	9,354 (9.72%)	2,224 (9.71%)	971 (11.36%)	1,146 (9.31%)
	Topic 9: Dislike vaping, especially the waste coming from disposable vaping products.	51,258 (10.27%)	9,664 (10.04%)	2,336 (10.20%)	768 (8.98%)	763 (6.20%)
	Total	499,106 (100%)	96,220 (100%)	22,895 (100%)	8,549 (100%)	12,305 (100%)

## Data Availability

Data will be made available on individual request.

## Abbreviations

API: Application Programming Interface
E-cigarette: Electronic cigarette
FDA: US Food and Drug Administration
LDA: Latent Dirichlet Allocation
RoBERTa: Robustly Optimized Bidirectional Encoder Representations from Transformers
MDOs: Marketing denial orders
